# Supplementary material for: Rising Lysophosphatidylcholine Levels Post-Hepatitis C Clearance
Source: Int J Mol Sci. 2024 Jan 18;25(2):1198. doi: 10.3390/ijms25021198 (PMC10816147; doi:10.3390/ijms25021198)
Supplement: Supplementary file 1 [file ijms-25-01198-s001.zip › ijms-2770260-supplementary.pdf]

Table S1: Spearman correlation coefficients for the correlation of LPC species with BMI and age of HCV patients before therapy. * p < 0.05. ** p < 0.01, *** p < 0.001.

**LPC nmol/ml**	**BMI kg/m²**	**Age Years**	**Age Years**	**Age Years**
	Whole cohort	Whole cohort	Patients without liver cirrhosis	Patients with liver cirrhosis
**15:0**	-0.109	-0.109	0.067	-0.195
**16:0**	-0.059	**-0.244***	-0.064	-0.283
**16:1**	-0.110	-0.088	-0.002	-0.152
**18:0**	0.004	-0.201	-0.028	-0.309
**18:1**	-0.162	-0.200	-0.127	-0.319
**18:2**	-0.122	**-0.313*****	-0.269	-0.226
**18:3**	-0.093	-0.204	-0.145	-0.241
**20:3**	-0.022	**-0.299*****	-0.127	-0.429
**20:4**	-0.033	**-0.337*****	-0.188	-0.221
**20:5**	-0.163	**-0.256****	-0.093	-0.190
**22:5**	-0.144	**-0.251****	-0.139	-0.211
**22:6**	-0.069	**-0.265****	-0.092	-0.189

Table S2: Spearman correlation coefficients for the correlation of LPC species with CRP, leukocytes and platelet count of patients without and with liver cirrhosis before therapy. * p < 0.05. ** p < 0.01.

	**Patients without liver cirrhosis**			**Patients with liver cirrhosis**		
**LPC nmol/ml**	**CRP mg/l**	**Leukocytes n/l**	**Platelets n/l**	**CRP mg/l**	**Leukocytes n/l**	**Platelets n/l**
**15:0**	-0.081	-0.157	0.080	-0.005	0.089	0.062
**16:0**	-0.091	-0.095	0.114	-0.075	0.023	0.141
**16:1**	-0.011	-0.048	0.055	-0.147	0.046	-0.059
**18:0**	-0.148	-0.162	0.085	0.001	0.074	0.233
**18:1**	-0.130	-0.028	0.144	-0.082	-0.166	-0.103
**18:2**	-0.119	-0.080	0.216	-0.041	-0.104	0.018
**18:3**	-0.098	0.011	**0.258***	-0.035	-0.169	0.014
**20:3**	-0.009	0.063	0.203	-0.180	0.194	0.179
**20:4**	0.108	-0.010	0.188	-0.184	0.134	0.152
**20:5**	0.069	0.056	**0.301****	-0.115	0.157	0.168
**22:5**	0.109	-0.066	0.085	-0.193	0.122	0.065
**22:6**	0.068	-0.110	0.172	-0.212	0.279	0.272

Table S3: Spearman correlation coefficients for the correlation of LPC species and viral load of patients with and without liver cirrhosis before therapy. * $p < 0.05$.

LPC	Viral load<br>Non-cirrhosis Patients	Viral load<br>Cirrhosis Patients
**15:0**	0.090	-0.084
**16:0**	0.089	-0.009
**16:1**	0.106	-0.093
**18:0**	0.093	0.034
**18:1**	0.166	0.026
**18:2**	0.137	-0.044
**18:3**	0.221	-0.115
**20:3**	0.091	0.042
**20:4**	-0.100	-0.045
**20:5**	0.073	0.029
**22:5**	-0.017	-0.079
**22:6**	-0.022	0.060

Table S4: Median, minimum and maximum values of LPC species in serum of patients with and without liver cirrhosis at therapy end.

LPC nmol/ml	No Cirrhosis			Cirrhosis			
	Median	Minimum	Maximum	Median	Minimum	Maximum	**p-value**
**15:0**	0.42	0.19	0.77	0.29	0.11	0.69	<0.001
**16:0**	38.09	18.46	58.61	26.51	9.12	55.79	<0.001
**16:1**	1.43	0.63	3.83	1.27	0.40	3.40	0.3574
**18:0**	12.31	4.92	22.10	8.70	3.25	18.01	<0.001
**18:1**	12.10	5.92	22.17	9.55	4.53	18.34	<0.001
**18:2**	15.85	6.94	43.11	12.18	4.24	24.07	<0.001
**18:3**	0.33	0.11	1.78	0.25	0.08	0.58	<0.001
**20:3**	1.27	0.53	3.28	0.83	0.25	1.92	<0.001
**20:4**	3.11	1.25	5.72	1.89	0.69	4.33	<0.001
**20:5**	0.28	0.10	2.09	0.15	0.06	0.52	<0.001
**22:5**	0.23	0.08	0.43	0.16	0.07	0.33	<0.001
**22:6**	0.73	0.29	2.08	0.42	0.09	0.75	<0.001
